# Benchmarking of Mutation Diagnostics in Clinical Lung Cancer Specimens

**DOI:** 10.1371/journal.pone.0019601

**Published:** 2011-05-05

**Authors:** Silvia Querings, Janine Altmüller, Sascha Ansén, Thomas Zander, Danila Seidel, Franziska Gabler, Martin Peifer, Eva Markert, Kathryn Stemshorn, Bernd Timmermann, Beate Saal, Stefan Klose, Karen Ernestus, Matthias Scheffler, Walburga Engel-Riedel, Erich Stoelben, Elisabeth Brambilla, Jürgen Wolf, Peter Nürnberg, Roman K. Thomas

**Affiliations:** 1 Max Planck Institute for Neurological Research with Klaus-Joachim-Zülch Laboratories of the Max Planck Society and the Medical Faculty of the University of Cologne, Cologne, Germany; 2 Laboratory of Translational Cancer Genomics, Center of Integrated Oncology Köln Bonn, University of Cologne, Cologne, Germany; 3 Cologne Center for Genomics, University of Cologne, Cologne, Germany; 4 Department I for Internal Medicine and Center of Integrated Oncology Köln-Bonn, University Hospital of Cologne, Cologne, Germany; 5 Institute for Pathology, Hospital Merheim, Cologne, Germany; 6 Max Planck Institute for Molecular Genetics, Berlin, Germany; 7 Pneumologische Gemeinschaftspraxis Dr. H.-P. Wagner-Dr. St. Klose-Dr. A. Kratzsch, Chemnitz, Germany; 8 Department of Medical Oncology, Lung Clinic Merheim, Cologne, Germany; 9 Department of Thoracic Surgery, Lung Clinic Merheim, Cologne, Germany; 10 Department of Pathology, Institut Albert Bonniot, INSERM U823, University Joseph Fourier, CHU Grenoble Hôpital Michallon, Grenoble, France; Charité Universitätsmedizin Berlin, NeuroCure Clinical Research Center, Germany

## Abstract

Treatment of *EGFR*-mutant non-small cell lung cancer patients with the tyrosine kinase inhibitors erlotinib or gefitinib results in high response rates and prolonged progression-free survival. Despite the development of sensitive mutation detection approaches, a thorough validation of these in a clinical setting has so far been lacking. We performed, in a clinical setting, a systematic validation of dideoxy ‘Sanger’ sequencing and pyrosequencing against massively parallel sequencing as one of the most sensitive mutation detection technologies available. Mutational annotation of clinical lung tumor samples revealed that of all patients with a confirmed response to *EGFR* inhibition, only massively parallel sequencing detected all relevant mutations. By contrast, dideoxy sequencing missed four responders and pyrosequencing missed two responders, indicating a dramatic lack of sensitivity of dideoxy sequencing, which is widely applied for this purpose. Furthermore, precise quantification of mutant alleles revealed a low correlation (r^2^ = 0.27) of histopathological estimates of tumor content and frequency of mutant alleles, thereby questioning the use of histopathology for stratification of specimens for individual analytical procedures. Our results suggest that enhanced analytical sensitivity is critically required to correctly identify patients responding to *EGFR* inhibition. More broadly, our results emphasize the need for thorough evaluation of all mutation detection approaches against massively parallel sequencing as a prerequisite for any clinical implementation.

## Introduction

Initially limited to rare tumors such as chronic myeloid leukemia (CML) or gastrointestinal stromal tumors (GIST), the success of treating mutationally activated kinases with kinase inhibitors has reached the group of the frequent “solid” tumors and has thereby profoundly changed clinical oncology. Treatment of patients suffering from *EGFR*-mutant cancers with *EGFR* inhibitors leads to objective response [1], [2], [3], to a doubling in progression-free survival as compared to standard chemotherapy and to long overall survival [4], [5]. Similar pairs of genomic lesions and targeted drugs are *BRAF* mutation and *BRAF* inhibition [6] *PARP* inhibition and *BRCA1/BRCA2* mutations [7], [8] or *PDGFRA*/*KIT* mutation and imatinib [9]. In light of the current efforts to systematically sequence the genomes of all major tumor types (http://www.icgc.org/ or www.cancergenome.nih.gov), the list of genetically defined tumors that are susceptible to a specific therapeutic intervention is likely to expand dramatically in the next few years. Together, these observations suggest a rapidly growing need for sensitive and accurate mutation detection in clinical specimens as part of routine clinical care.

Unfortunately, despite appearing trivial, clinical implementation of mutation detection encounters both sample-related and methodological problems: first, the majority of clinical samples available are small-sized, formalin-fixed and paraffin-embedded (FFPE) biopsies or cytological specimens typically yielding limited amounts of low quality DNA, all of which seriously affect PCR amplification and subsequent analyses. Furthermore, the tumor composition as well as multiple types of non-neoplastic cells affect the detection of tumor-specific somatic mutations in a background of non-mutant, “wild-type” alleles. Second, conventional sequencing approaches lack sensitivity for the detection of such rare alleles.

Several methods were established to offer sensitive mutation detection, all including DNA extraction, PCR amplification and subsequent mutation analysis by sequencing or genotyping-based assays [10], [11], [12], [13], [14], [15], [16], [17], [18], [19]. In several instances, enrichment of tumor cells is achieved prior mutation detection, e.g., by laser-assisted microdissection of tumor specimens [15], [17]. Unfortunately, there is an almost universal lack of validation of clinically relevant mutation detection approaches in clinical settings against a sensitive gold standard approach.

We have recently introduced massively parallel sequencing for mutation detection in clinical cancer specimens [20]. Such approaches are widely considered to provide the highest sensitivity currently available for this purpose due to the ability to sample rare mutant alleles in a predominant background of wild-type alleles in any tumor specimen [20]. Furthermore, this approach is not restricted to an a-priori selection of mutations considered relevant. Therefore, massively parallel sequencing is ideally suited to validate other mutation detection methodologies.

In this study, we have validated two sequencing-based mutation detection approaches (dideoxy- and pyrosequencing) against parallel sequencing in a clinical setting, taking into account the tumor cell content as well as the quality and the type of tissue (e.g. FF, FFPE) of each specimen.

## Materials and Methods

### Ethics Statement

This study was approved by the Ethics Committee of the Faculty of Medicine of the University of Cologne and all patients gave written informed consent. A subgroup of patients (n = 9) participated in the ERLOPET trial (NCT00568841).

### Tumor samples and cell lines

In this study, we have analyzed 24 tumor samples obtained from 22 patients (**Table 1**). This study was approved by the local Ethics Committee and all patients gave written informed consent. A subgroup of patients (n = 9) participated in the ERLOPET trial (NCT00568841). Tumor specimens were reviewed by a reference pathologist to determine the tumor-cell content for subsequent dissection of areas with highest tumor-cell content. NSCLC cell lines harbouring different *EGFR* and *KRAS* mutations were obtained and cultured as described previously (**Supplementary Table S1**) [21]. Genomic DNA of all tumor specimens and cell lines was extracted using Gentra Pure Gene Tissue Kit (Qiagen) followed by DNA quantification using Picogreen dsDNA reagents (Invitrogen).

**Table 1:** Clinical characteristics and genomic alterations in NSCLC specimens.

case	histotype	sex	age	tissue type	specimen type	tumor content (%)	allel (%)	TKIresp.	dideoxy	pyro	parallel	mutation
01	AD	M	38	FF	wedge Bx	80	N/A	SD	WT	WT	WT	no mutation
14	SQ	M	67	FFPE	transbronchial Bx	95	N/A	SD	WT	WT	WT	no mutation
21	SQ	M	39	FFPE	Bx of chest wall	90	N/A	PD	WT	WT	WT	no mutation
26	AD	M	60	H&E	pleural effusion	5	N/A	PD	WT*	WT*	WT	no mutation
28	AD	F	69	FF	lung surgery	70	N/A	N/A	WT	WT	WT	no mutation
33	AD	M	61	FFPE	transbronchial Bx	80	N/A	N/A	WT	WT	WT	no mutation
34	AD	M	44	FFPE	lung surgery	80	N/A	N/A	WT	WT	WT	no mutation
02	AD	F	69	FFPE	CT-guided lung Bx	50	54	PR	MUT	MUT	MUT	EGFR exon 19 E746_A750del (Del-1a)
03	AD	M	58	FFPE	cerebral surgery	80	30	PR	MUT	MUT	MUT	EGFR exon 21 L858R
04	SQ	M	58	FFPE	transbronchial Bx	80	58	PR	MUT	MUT	MUT	EGFR exon 19 E746_T751del, S752V
05	AD	M	76	FFPE	mediastinoscopy	50	11	PR	WT	MUT	MUT	EGFR exon 19 E746_R748del,A750P
06a)	AD	M	40	FF	cervical LN Bx	70	36	PR	MUT	MUT	MUT	EGFR exon 19 E746_A750del (Del-1b)
06b)	AD	M	40	CSF	lumbar puncture	N/A	68	RL	MUT	MUT	MUT	EGFR exon 19 E746_A750del (Del-1b)
08	AD	F	52	FFPE	supraclavicular LN Bx	80	49	PR	MUT	MUT	MUT	EGFR exon 19 E746_A750del (Del-1b)
10b)	AD	F	63	FF	Liver Bx	80	95	PR	MUT	MUT	MUT	EGFR exon 19 L747_S752del, P753S
10b)	AD	F	63	FF	Liver Bx	80	20	RL$	WT	MUT	MUT	EGFR exon 20 T790M
12	AD	F	80	FF	lung sugery	90	52	PR	MUT	MUT	MUT	EGFR exon 19 E746_A750del (Del-1a)
13a)	AD	M	62	H&E	pleural effusion	5	8	PR	WT	WT	MUT	EGFR exon 19 L747_A750del, T751P
13b)	AD	M	62	FF	pleural effusion	70	74	RL	MUT	MUT	MUT	EGFR exon 19 L747_A750del, T751P
27	AD	F	71	FF	CT-guided lung Bx	40	11	PR	WT	MUT	MUT	EGFR exon 19 E746_A750del (Del-1a)
31	AD	F	66	FF	transbronchial Bx	60	6	PR	WT	WT	MUT	EGFR exon 19 E746_A750del (Del-1b)
11	AD	M	55	FFPE	lung surgery	50	21	N/A	WT	MUT	MUT	KRAS exon 2 G12A
19	AD	F	52	FFPE	lung surgery	50	36	SD	MUT	MUT	MUT	KRAS exon 2 A11V; G12V
24	AD	M	69	FFPE	lung surgery	95	40	PD	MUT	MUT	MUT	KRAS exon 2 G13C
30	AD	F	61	FFPE	lung surgery	35	29	PD	MUT	MUT	MUT	KRAS exon 2 G12V

Abbreviations: AD, adenocarcinoma; Bx, biopsy; CSF, cerebral-spinal fluid; Del, deletion in *EGFR* exon 19 (Del-1a: E746_A750_2235–2249del; Del-1b: E746_A750_2236–2250del); F, female; FF, Fresh-Frozen; FFPE, Formalin-fixed, paraffin-embedded; H&E, haematoxylin and eosin-stained tumor section; M, male; LN, lymph node; N/A, not applicable; MUT, mutation; PD, progressive disease; PR, partial remission; RL, relapse; SD, stable disease; SCLC, small cell lung cancer; SQ, squamous cell carcinoma; TKI, tyrosine kinase inhibitor; WT, wild-type *EGFR* and *KRAS*;

a) Tumor specimen obtained prior to TKI treatment.

b) Tumor specimen obtained after relapse from TKI treatment.

*Mutation detection sensitivity of dideoxy and pyrosequencing is limited due to rare tumor cell content possibly generating false-negative samples.

$ Case 10 achieved a partial response to TKI treatment. However, the analyzed tumor specimen was obtained at the time of relapse and is therefore indicated as PR (partial remission) and RL (relapse).

### PCR and direct dideoxy sequencing

*EGFR* exon18–21 and *KRAS* exon 2 and 3 were amplified by nested PCR and gene specific external and internal primer pairs (**Supplementary Table S2**). Internal primers are equipped with the M13-primer sequence to facilitate sequencing. For external PCR reactions, we used 5–10 ng (40–60 ng low quality DNA) of genomic DNA. PCR reactions were carried out in a total volume of 50 µl (0.2 µM of each primer, 2 mM MgCl_2_, 200 µM of each dNTP and 1.25 U HotStarTaq *Plus* DNA Polymerase KIT (Qiagen)) and the following cycling conditions: 95°C for 5 min, 30 cycles at 95°C for 30 sec, 60°C for 30 sec and 72°C for 1 min and final extension at 72°C for 10 min. After ExoSAP-IT (USB Corporation) treatment PCR products were bidirectional sequenced using the M13-sequencing primers and BigDye Terminator Mix version 3.1 (Applied Biosystems) on ABI 3730 (Applied Biosystems). Sequencing electropherograms were analyzed by visual inspection of electropherograms and by Mutation Surveyor 2.03 Software (SoftGenetics). Fragment length analysis of *EGFR* exon 19 was carried out by standard PCR (For-Hex-ctggatccagaaggtgaga and Rev-ccacacagcaaagcagaaac) resulting in amplicons of 118-bp (Ta = 59°C) for wild-type exon 19. PCR products were analyzed on ABI 3700 DNA sequencer and scored by GeneMarker software (SoftGenetics).

### Pyrosequencing assays

We have designed five different pyrosequencing [22] assays for sequencing analysis of *EGFR* exon 19–21 and *KRAS* exon 2 and 3 (**Supplementary Table S3**). Template DNA (6 ng genomic DNA or external PCR products) was amplified using HotStarTaq *Plus* DNA Polymerase KIT (Qiagen) and standard protocol (0.2 µM of each primer, 160 mM dNTPs, 2 U enzyme) and cycling conditions (45 cycles and Ta = 59°C for *EGFR* exon 19, Ta = 60°C for *KRAS* exon 2, Ta = 63°C for *EGFR* exon 20, Ta = 61°C for *EGFR* exon 21 and *KRAS* exon 3 and Ta = 60°C for *KRAS* exon 2). Reverse PCR primers were biotinylated for subsequent pyrosequencing analysis. Pyrosequencing reactions were carried on PSQ HS96A instrument using PSQ HS96A SNP reagents and pyrosequencing SNP analysis software (Biotage AB, Uppsala, Sweden, now named PyroMark™Q96MD by Qiagen). For sensitivity studies, quantified PCR products of *EGFR*-mutant cells or *KRAS*-mutant cells were diluted with wild-type *EGFR* or wild-type *KRAS* amplicons, respectively. Pyrosequencing raw data signals were normalized using known wild-type signals. Significant mutation calling was determined from experimental noise by statistical analysis of raw data using T-test (p≤0.05).

### Massively parallel sequencing analysis

Massively parallel sequencing was performed using the GS FLX Standard or GS FLX Titanium chemistry according to standard protocols (Roche). Amplicon libraries were generated using external PCR products as templates and standard protocols and conditions of FastSTart Hifi PCR system (Roche) (**Supplementary Table S4**). We used on average 340.000 beads per run yielding 80.000–120.000 reads in total and an amplicon coverage ranging from 600× to 1500× with an average amplicon length of 250–300 bp covering the entire exon. All reads were aligned to chromsome 7 and 12 of the reference genome (hg18) using BWA [23]. Finally, aligned reads were visualized and analyzed by the Integrative Genomics Viewer (http://www.broadinstitute.org/igv/). The Students T test was used for comparison of relative read frequency between *EGFR* mutated and wildtype tumors. We set the sequencing error rate to 0.1% in areas without homopolymers [24]. The threshold for significant detection of mutated alleles was defined using a Poisson distribution of the known error rate taking into account the coverage leading to a detection limit between 0.3 and 0.5% mutant alleles.

## Results

### Patients and tumor specimens

From September 2008 to April 2009, we have analyzed patients with histologically confirmed NSCLC for the presence of mutations in exons 18–21 of *EGFR* and exons 2 and 3 of *KRAS*. Patients enrolled in this mutation analysis study are not representative for the prevalence of such mutations in NSCLC as they were enriched for cases with an increased likelihood of being *EGFR* mutation-positive based on histological and clinical features (e.g., lung adenocarcinoma in never or light ex-smokers). In this benchmarking study, we have analyzed 24 tumors from 22 patients (**Table 1**) for a systematic comparison of mutation detection performance of conventional dideoxy- and pyrosequencing against one of the most sensitive sequencing technology, massively parallel sequencing [20]. The tumor-cell content of each of these specimens was estimated by a pathologist and ranged from 5% to up to 95%. Clinical specimens comprised formalin-fixed and paraffin-embedded (FFPE) tumor tissue blocks as well as fresh-frozen (FF) biopsies or cytological slides obtained from pleural effusions (**Table 1**).

### Detection of clinically relevant mutations by methods of different sensitivity

**Figure 1:**
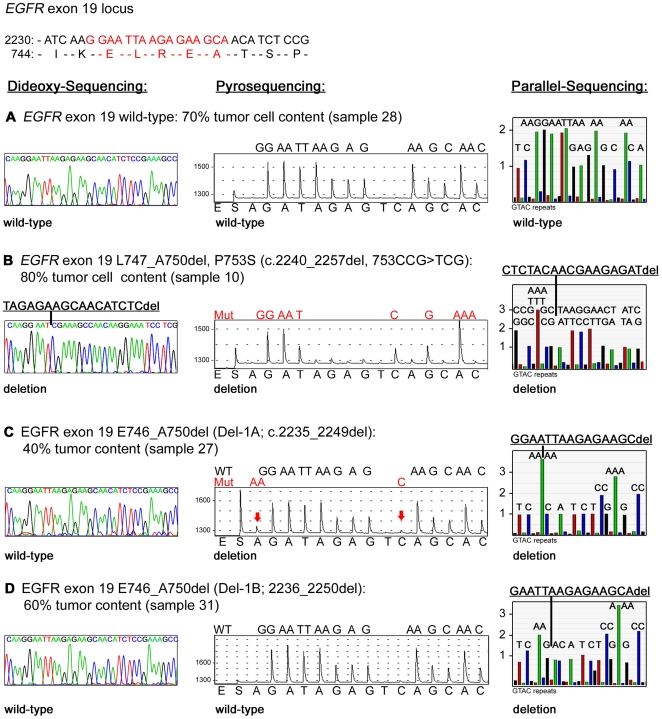
*EGFR* exon 19 mutation analysis in NSCLC tumor samples. Sequencing analysis of *EGFR* exon 19 was performed by dideoxy sequencing (electropherograms in the left panels), pyrosequencing (pyrogams in the middle panels) and massively parallel sequencing (programs in right panel). (A) Wild-type *EGFR* exon 19 detected by all three sequencing techniques; (B) L747_A750del, P753S mutation detected by all three sequencing techniques; (C) E746_A750del (Del-1A) mutation identified by pyrosequencing and massively parallel sequencing; (D) E746_A750del (Del-1B) only detected by massively parallel sequencing. del, deletion; Mut, mutation; WT, wild-type. Arrows indicate position of expected mutation specific signals.

We first performed conventional dideoxy-nucleotide chain termination-based (“Sanger”) sequencing on all of these specimens because, despite its limitations, this method is still the most widely used approach for mutation detection in clinical specimens. We determined the sensitivity of this sequencing technique to range from 20–30% of mutated alleles by mixing PCR products of *KRAS*-mutant and wild-type cell lines (**Supplementary Fig. S1**). Mutation screening of 24 tumor samples by dideoxy sequencing revealed short in-frame nucleotide deletions in *EGFR* exon 19 (**Table 1** **,** **Fig. 1A and 1B**) in eight samples and only one sample (case 03) harbored the L858R point mutation in exon 21 of *EGFR* (**Table 1**). Moreover, three samples (case 19, 24 and 30) had mutations in exon 2 of the *KRAS* gene (**Table 1**).

We next tested whether conventional pyrosequencing [22] was able to confirm mutations found by dideoxy sequencing and if additional mutations could be found by this method in the patient cohort tested. Pyrosequencing offers cost-effective quantitative detection of sequence variants and was previously shown to enable sensitive *KRAS* mutation detection in colorectal cancer [12], [25], [26]. The enhanced sensitivity for detection of rare variants is due to the generation of non-interfering, individual signals for mutant and wild-type alleles. We established sensitive (5–10% mutant allele), reproducible and linear pyrosequencing assays for the most prominent mutation hotspots in *EGFR* and *KRAS* (**Supplementary Fig. S2, S3, S4, S5, S6, S7, S8, S9, S10**). Pyrosequencing confirmed all mutations detected by dideoxy sequencing (**Table 1**). However, we also detected four additional mutations in our sample cohort that had been missed by dideoxy sequencing (**Table 1**, **Fig. 1C** and **Supplementary Fig. S3A, S6B, S9A**). For example, we detected the erlotinib resistance mutation, T790M, in tumor sample 10 (80% tumor cell content) obtained at the time of relapse (**Supplementary Fig. S6B**) and this sample also harbored the L747_S752del_P753S deletion initially detected by dideoxy sequencing (**Table 1** **and Fig. S4C**). We further found a previously undetected G12A substitution in exon 2 of *KRAS* in sample 11 (50% tumor cell content, **Supplementary Fig. S9A**) and confirmed this mutation by subcloning of *KRAS* exon 2 amplicons and subsequent dideoxy sequencing (data not shown). *EGFR* exon 19 pyrograms of sample 27 (40% tumor cells) and of sample 05 (50% tumor cells) exhibited fluorescence signals indicative for the presence of mutations that were significantly above the level of experimental noise (**Fig. 1C** **and Supplementary Fig. S3A**). Fragment-length analyses of *EGFR* exon 19 PCR products validated this deletion in sample 27 **(Supplementary Fig. S11**).

Finally, we employed massively parallel array-based pyrosequencing-by-synthesis [20] to validate mutation results obtained by conventional dideoxy and pyrosequencing. We sequenced all of the 24 specimens to an average coverage of 1079× and a minimum coverage of 600× per exon and specimen. Data analysis confirmed the presence of *EGFR* and *KRAS* mutations in 13 samples initially identified by both, dideoxy sequencing and pyrosequencing (**Table 1**). Furthermore, massively parallel sequencing also validated the four additional mutations that were newly detected by sensitive pyrosequencing: *EGFR* exon 19 deletions in sample 05 and 27, the G12A mutation of *KRAS* in sample 11 and the T790M mutation of *EGFR* in sample 10 (**Table 1**). The parallel nature of this sequencing approach enabled precise quantification and identification of these low-frequency variants. Specifically, we identified the exon 19 mutation in sample 05 to be a 9-bp in-frame deletion encoding the amino acid deletion-substitution E746_R748del_A750, at a frequency of 11% of 1315 reads (**Table 1**). Furthermore, we quantified a 12-bp deletion (E746_A750) to occur at a frequency of 11% of 854 reads in sample 27 (**Fig. 1C**). The G12A substitution in sample 11 was present in 21% of 1358 reads and the T790M resistance mutation in sample 10 occurred at a frequency of 20% out of 909 reads (**Table 1**).

In addition to validating the previously detected mutations parallel sequencing identified *EGFR* exon 19 deletions in sample 31 and 13a, which were indistinguishable from experimental noise in both conventional dideoxy and pyrosequencing analyses. Parallel sequencing enabled the detection of an E746_A750del in sample 31 at a frequency of 6% of 1081 reads (**Table 1** and **Fig. 1D**). Remarkably, this specimen had been estimated to contain 60% tumor cells by histopathology (**Table 1**). Similarly, flowgrams of sample 13a (5% tumor cells) revealed a 12-bp deletion (L747_A750del_T751P) at a frequency of approximately 8% of 658 reads (**Table 1**). This mutation was identical to the one already detected by dideoxy sequencing in another specimen of the same patient (samples 13b) with higher tumor content (70%), obtained at the time of relapse (**Table 1**). Thus, beyond validating all of the other mutations that had been called by dideoxy sequencing (n = 12) and pyrosequencing (n = 16), massively parallel sequencing identified two further mutated samples that had been missed by the other two methods due to insufficient sensitivity (**Table 1** **and Supplementary Table S5**).

We next analyzed high-quality 454 sequencing reads (phred score >30) with the goal of detecting T790M mutations occurring at low frequency in specimens obtained before therapy. Exon 20 was covered to an averaged depth of 1018 reads in the region of codon 790. In patients whose tumors had a deletion in exon 19 or L858R we observed a significantly higher (p = 0.03) number of reads containing the T790M mutation as compared to patients with wildtype *EGFR* (EGFR mut: mean = 2.5/1000; range 0–7.5/1000; EGFR wt: mean 0.8/1000 (0–2.3/1000). We note that these allele frequencies are in the range of the technological limit of accuracy. However, the number of reads with T790M was above the threshold for mutation calling in 27% of the samples with deletion in exon 19 or L858R but in no sample without *EGFR* mutation (**Supplementary Fig. S12**).

Taken together, massively parallel sequencing identified a total of 18 mutations in *EGFR* and *KRAS* resulting in a sensitivity of 67% for dideoxy sequencing and 89% for pyrosequencing (**Table 1** **and Supplementary Table S5**). Despite the limited size of our sample set, these results underscore the dramatic lack of sensitivity of dideoxy sequencing in clinical mutation detection.

### Sensitivity of mutation detection as a function of tumor cell content

**Figure 2:**
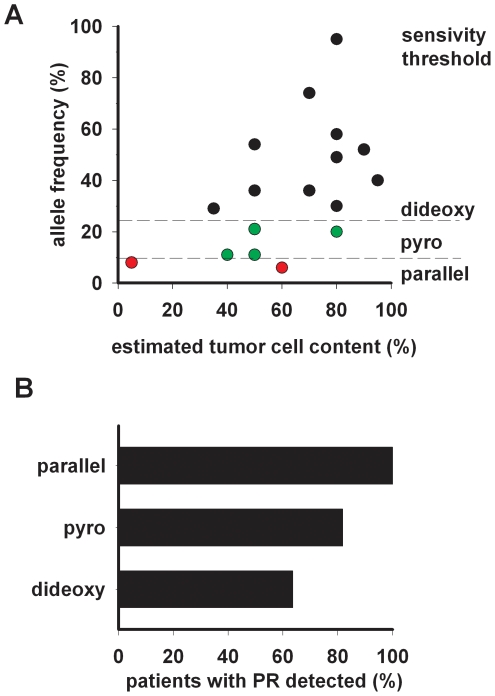
Mutation detection performance in NSCLC tumor samples using dideoxy sequencing, pyrosequencing and massively parallel sequencing. (A) Correlation between the estimated tumor cell content and the actual frequency of mutated alleles determined by massively, parallel sequencing data. Black, mutations detected by dideoxy and pyrosequencing; Green, mutations detected by pyrosequencing, but missed by dideoxy sequencing; Red, mutations only detected by parallel sequencing. (B) Sensitivity of dideoxy sequencing, pyrosequencing and massively parallel sequencing in patients with confirmed clinical response to treatment with erlotinib.

Tumor-cell content is a critical parameter for the performance of mutation detection in cancer [20] and is the basis for microdissection-based tumor-cell enrichment. We therefore sought to analyze the performance of the three mutation analysis methods as a function of histopathologically estimated tumor-cell content. The mean tumor content of samples identified as being mutant by dideoxy sequencing was 78% (range 35–95%) and 71% (range 35–95%) in the case of pyrosequencing (**Fig. 2A**). All samples, in which dideoxy sequencing had missed mutations, contained 80% tumor cells or less. By contrast, the specimens in which pyrosequencing had missed mutations had 60% tumor content or less (**Fig. 2A**).

We next assessed the correlation between tumor-cell content and frequency of mutated alleles as determined by massively parallel sequencing (**Fig. 2A**). We observed a low correlation between the analytically determined allele frequency and the tumor-cell content (*r^2^* = 0.27, *p* = 0.029). Remarkably, the detection limits of the different approaches determined by counting of mutant and wild-type alleles detected by massively parallel sequencing in the primary tumors were highly similar to those observed in the initial allele mixing experiments (**Supplementary Figs. S2, S3, S4, S5, S6, S7, S8, S9, S10**): 29% for dideoxy sequencing and 11% for pyrosequencing (**Table 1** **and** **Fig. 2A**). The allele frequencies of mutations found by pyrosequencing but not dideoxy sequencing ranged between 11% and 21% (**Fig. 2A**). Mutations only detected by massively parallel sequencing occurred at allele frequencies of less than 10% (**Table 1** and **Fig. 2A**). Strikingly, the tumor content in these cases had been estimated to be 60% and 5%, respectively. Thus, histopathological estimates of tumor-cell content are not predictive of the frequency of mutant alleles, while the ability to detect mutations is limited by the allele frequency but not by the tumor cell content.

### EGFR mutations and clinical outcome

Previous analyses of the association of *EGFR* mutations and clinical benefit induced by *EGFR* inhibition has occasionally yielded inconsistent results in that some studies have reported a lack of such association [27], [28], [29] Such inconsistencies might result from the failure to detect mutations with sufficient sensitivity and accuracy. Importantly, in our cohort, only massively parallel sequencing was able to detect a sensitizing *EGFR* mutation in *all* patients that had experienced a partial response (according to RECIST criteria) following treatment with erlotinib (**Fig. 2B**). By contrast, only 7 out of 11 patients with confirmed PR were detected by dideoxy sequencing and 9 of 11 were identified by pyrosequencing (**Fig. 2B**). Thus, despite the limited size of our patient cohort, these results support the notion that the dramatic differences in sensitivity strongly skew the association between the presence of *EGFR* mutations and response to erlotinib. Patients with *EGFR*-wild-type tumors and *KRAS*-mutant tumors who had received erlotinib exhibited stable or progressive disease (**Table 1**) in accordance with previous reports [30].

## Discussion

Here, we show how the limited sensitivity of methods that are widely applied to clinical mutation diagnostics could lead to a critical inaccuracy in genetic patient stratification. In particular, the low sensitivity of dideoxy sequencing led to misdiagnoses in 6 of 24 lung cancer specimens. If the presence of an *EGFR* mutation had been the inclusion criterion for treatment with *EGFR* inhibitors, four patients would not have received the right treatment. Thus, despite the limited size of our sample set, these results underscore the inadequacy of dideoxy sequencing for clinical cancer gene mutation diagnostics. By contrast, conventional pyrosequencing offered enhanced sensitivity with only two patients being misdiagnosed. Furthermore, massively parallel sequencing robustly and accurately identified all mutations present in the dataset. We further show that parallel sequencing can detect preexisting T790M mutations in a 27% fraction of *EGFR*-mutant patients [31]. These results support the conclusion that massively parallel sequencing is the most sensitive technology currently available in clinical mutation diagnostics and suggest that pyrosequencing might be an alternative to dideoxy sequencing.

While many techniques have emerged over the past few years to match the ever-growing need to provide accurate clinical mutation analyses to oncologists, none of these have been systematically benchmarked against massively parallel sequencing, the method with the highest sensitivity for clinical mutation detection to date. Extrapolating differences such as those observed in this study to the large number of cancer patients worldwide indicates that many patients are being misdiagnosed each year. Thus, carefully developed diagnostic approaches (including thorough validation against massively parallel sequencing) will help targeting effective treatment to the right patient population.

Microdissection has been applied widely to increase the fraction of tumor cells in a given specimen in order to enhance the sensitivity of dideoxy sequencing. Identification of tumor-rich areas is a prerequisite for such procedure. We found, however, only a low correlation of tumor-cell content and frequency of mutant alleles. We assume that a routine survey of histopathological sections cannot accurately estimate the degree of “contamination” of the mutant allele by healthy bystander cells (e.g. normal lung tissue), that obscure the frequency of the mutant allele. Thus, microdissection can only partially rescue the limited sensitivity of dideoxy sequencing and should therefore also be validated carefully against massively parallel sequencing.

Another frequently discussed topic is the question of whether genotyping or sequencing might be the optimal strategy for clinical mutation detection in cancer. Even though our results suggest that pyrosequencing is a sensitive, accurate and cost-effective method to analyze the vast majority of samples with a tumor content of 20–70%, other methods, including those based on genotyping, might perform equally well. In case of already known hot-spot mutations locked nucleic acid (LNA) approaches are able to detect mutant alleles occurring at frequencies as low as 0.1% [14], [16]. Other techniques that may have value in clinical application include immunohistochemistry using *EGFR* mutation-specific antibodies [32] or the application of molecular beacons coupled by fluorescent imaging [33]. However, it is important to keep in mind that the intrinsic inability of genotyping methods to cover all clinically relevant mutations, adds to any analytical insensitivity. Based on these considerations, we favor sequencing-based mutation detection over genotyping. Although our sample size is too limited to comprehensively calculate sensitivity and specificity of mutation detection, we believe that the striking inferiority of conventional Sanger sequencing to detect therapeutically relevant oncogene mutations is of considerable interest to molecular pathologists and clinical oncologists.

In summary, we have shown how sequencing technologies with inferior sensitivity may fail to detect clinically relevant oncogene mutations in cancer patients. We therefore conclude that any novel method for clinical mutation diagnostics should be thoroughly validated against massively parallel sequencing in order to provide a correct genetic analysis as basis for molecularly targeted therapy.

## Supporting Information

Figure S1 **Sensitivity study of dideoxy sequencing and Pyrosequencing.** Different mixtures of PCR products from wild-type or G12S mutant NSCLC cell lines were used to determine the sensitivity limit of dideoxy sequencing (∼20–30%) and pyrosequencing (∼5%). Mutation specific signals are marked by asterisks in dideoxy electropherograms (left panels) and red arrows in programs (right panels). Mut, mutation; WT, wild-type.
(TIF)

Figure S2 **Sensitivity and linearity testing of the** ***EGFR*** **exon 19 pyrosequencing assay.** Mixtures of E746_A750del (Del-1a) mutant and wild-type PCR products of NSCLC cell lines were used to analyse the mutation detection limit and assay linearity. The assay is sensitive to a minimum of 5 to 10% of mutated alleles. Mutation specific signals are marked by red arrows. del, deletion; Mut, mutation; WT, wild-type.
(TIF)

Figure S3 **Pyrosequencing analysis of** ***EGFR*** **exon 19 in NSCLC tumor samples.** (A) *EGFR* exon 19 deletion identified in sample with 50% tumor cell content that was previously not identified by dideoxy sequencing; (B) No significant mutation detection in sample 13a with a 5% tumor cell content. Expected position of mutation specific signals is marked by red arrow. del, deletion; Mut, mutation; WT, wild-type.
(TIF)

Figure S4 **Pyrograms of** ***EGFR*** **exon 19 mutant NSCLC cell line and tumor samples.** (A) Del-B mutation in cell line HCC827; (B–D) Tumor specimens with an high tumor cell content enabling precise characterisation of the individual mutation; (E, F) Tumor specimens with a moderate (50% and 80%) tumor cell content resulting in overlapping signals of wild-type and mutant alleles. Mutation specific signals are marked by red arrows. del, deletion; Mut, mutation; WT, wild-type.
(TIF)

Figure S5 **Sensitivity and linearity testing of the** ***EGFR*** **exon 21 (L858R) pyrosequencing assay.** (A) Mixture study of PCR products from wild-type and L858R mutant NSCLC cell lines to determine the assay sensitivity limit of 5%–10%; (B) L858R point mutation identified in sample 03 (80% tumor cell content). Mutation specific signals are marked by red arrows. Mut, mutation; WT, wild-type.
(TIF)

Figure S6 **Sensitivity and linearity testing of the** ***EGFR*** **exon 20 (T790M) pyrosequencing assay.** (A) Mixture study of PCR products from wild-type and T790M mutant NSCLC cell lines to determine the assay sensitivity limit of 5%–10%; (B) T790M point mutation detected in sample 10 (80% tumor cell content) that was previously not detected by conventional dideoxy sequencing. Mutation specific signals are marked by red arrows. Mut, mutation; WT, wild-type.
(TIF)

Figure S7 **Sensitivity and linearity testing of the** ***KRAS*** **exon 2 (G12X;G13X) pyrosequencing assay.** Mixture study of PCR products from wild-type and G12S *KRAS* exon 2 mutant NSLC cell lines to determine the assay sensitivity limit 0f 5%–10%. Mutation specific signals are marked by red arrows. Mut, mutation; WT, wild-type.
(TIF)

Figure S8 **Pyrograms of** ***KRAS*** **exon 2 mutant NSCLC cell lines.** (A) wild-type *KRAS* in H1650; (B) G12D in SKLU1; (C) G12V in H2887; (D) G13C in H1355; (E) G12C in H2122; (F) G12A in H2009. WT, wild-type.
(TIF)

Figure S9 **Pyrograms of NSCLC tumor samples harbouring different** ***KRAS*** **mutations.** (A) G12A in sample 11; (B) A11V, G12V in sample 19; (C) G13C in sample 30 and (D) G12V in sample 30. Mut, mutation; WT, wild-type.
(TIF)

Figure S10 **Sensitivity and linearity testing of the** ***KRAS*** **exon 3 (Q61X) pyrosequencing assay.** Mixture study of PCR products from wild-type and Q61H *KRAS* exon 3 mutant NSCLC cell lines to determine the assay sensitivity limit of 10%. Mutation specific signals are marked by red arrows. Mut, mutation; WT, wild-type.
(TIF)

Figure S11 **Fragment length analysis of** ***EGFR*** **exon 19 PCR products.** (A) sample 27 harbouring a 15 bp deletion (100 bp fragment); (B) wild-type *EGFR* exon 19 (115 bp fragment) of sample 28.
(TIF)

Figure S12 **Read frequency of T790M mutation in pre-treatment tumor specimens.** Depicted is the average read frequency of T790M separately for *EGFR* wildtype and *EGFR* mutated tumor specimens. Tumor specimen 10b with a high allele frequency of T790M and clinical resistance to erlotinib treatment is not shown in this diagram.
(PPT)

Table S1 **EGFR and KRAS mutant NSCLC cell lines.** NSCLC cell lines harbouring different EGFR and KRAS mutations with respective amino acid changes are shown.
(DOC)

Table S2 **Primer sequences for EGFR and KRAS nested PCR.** External and internal primer sequences used for EGFR exon18–21 and KRAS exon 2 and 3 PCR are shown.
(DOC)

Table S3 **Primer sequences for EGFR and KRAS pyrosequencing.** Shown are primer sequences for five different pyrosequencing assays for sequencing analysis of EGFR exon 19–21 and KRAS exon 2 and 3.
(DOC)

Table S4 **EGFR and KRAS gene specific primers for massively parallel sequencing.** Primer pairs for massively parallel sequencing of EGFR exon 18–21 and KRAS exon 2 and 3 are depicted.
(DOC)

Table S5 **Comparison of mutation detection performance between sequencing methods.** Sensitivity of EGFR and KRAS mutation detection by three different sequencing technologies (dideoxy sequencing, pyrosequencing, parallel sequencing) is shown.
(DOC)
