# Supplementary figures and images for: Benchmarking of Mutation Diagnostics in Clinical Lung Cancer Specimens
Source: PLoS One. 2011 May 5;6(5):e19601. doi: 10.1371/journal.pone.0019601 (PMC3088700; doi:10.1371/journal.pone.0019601)

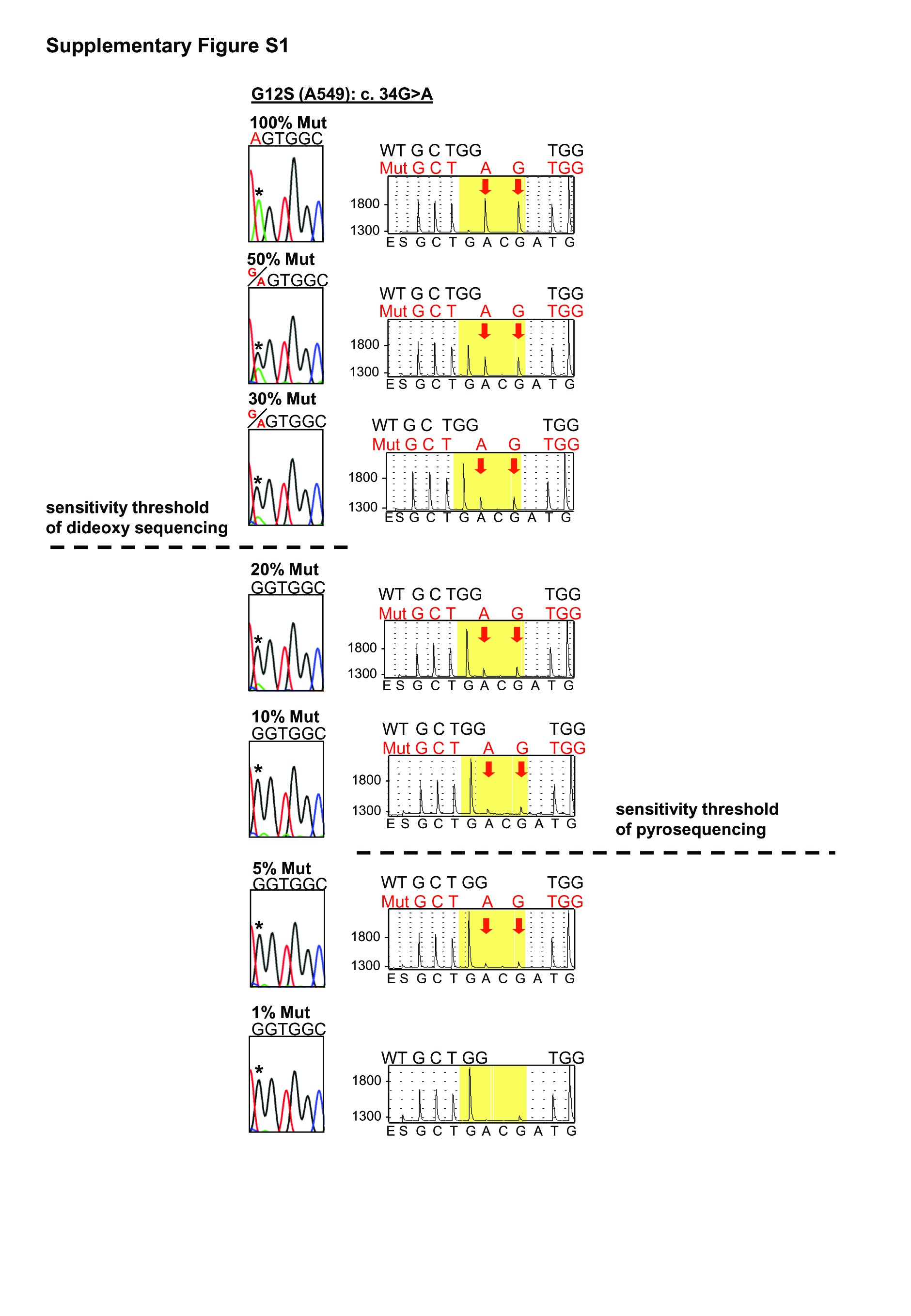

Supplement: Figure S1 — Sensitivity study of dideoxy sequencing and Pyrosequencing. Different mixtures of PCR products from wild-type or G12S mutant NSCLC cell lines were used to determine the sensitivity limit of dideoxy sequencing (∼20–30%) and pyrosequencing (∼5%). Mutation specific signals are marked by asterisks in dideoxy electropherograms (left panels) and red arrows in programs (right panels). Mut, mutation; WT, wild-type. (TIF) [file pone.0019601.s001.tif]

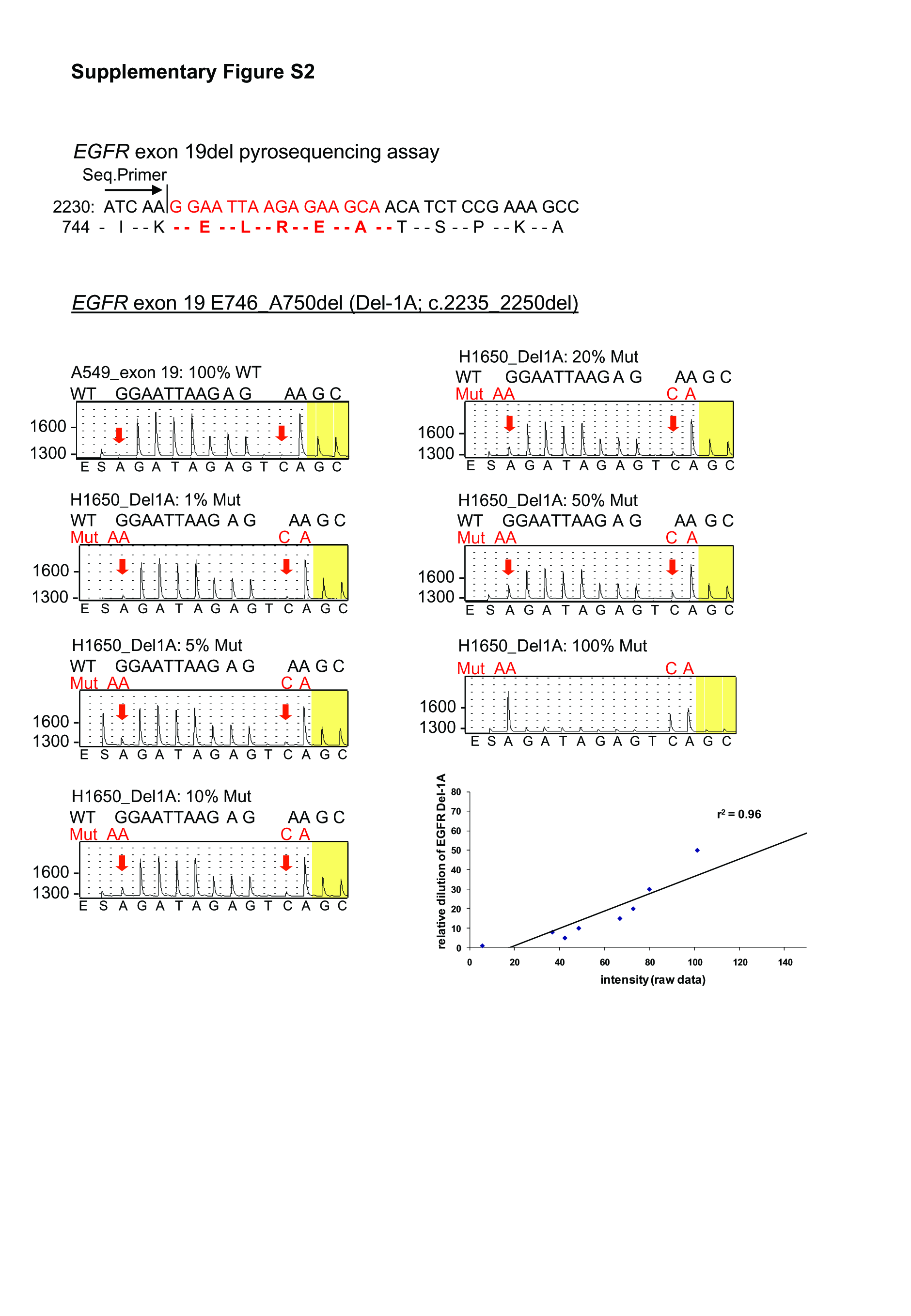

Supplement: Figure S2 — Sensitivity and linearity testing of the EGFR exon 19 pyrosequencing assay. Mixtures of E746_A750del (Del-1a) mutant and wild-type PCR products of NSCLC cell lines were used to analyse the mutation detection limit and assay linearity. The assay is sensitive to a minimum of 5 to 10% of mutated alleles. Mutation specific signals are marked by red arrows. del, deletion; Mut, mutation; WT, wild-type. (TIF) [file pone.0019601.s002.tif]

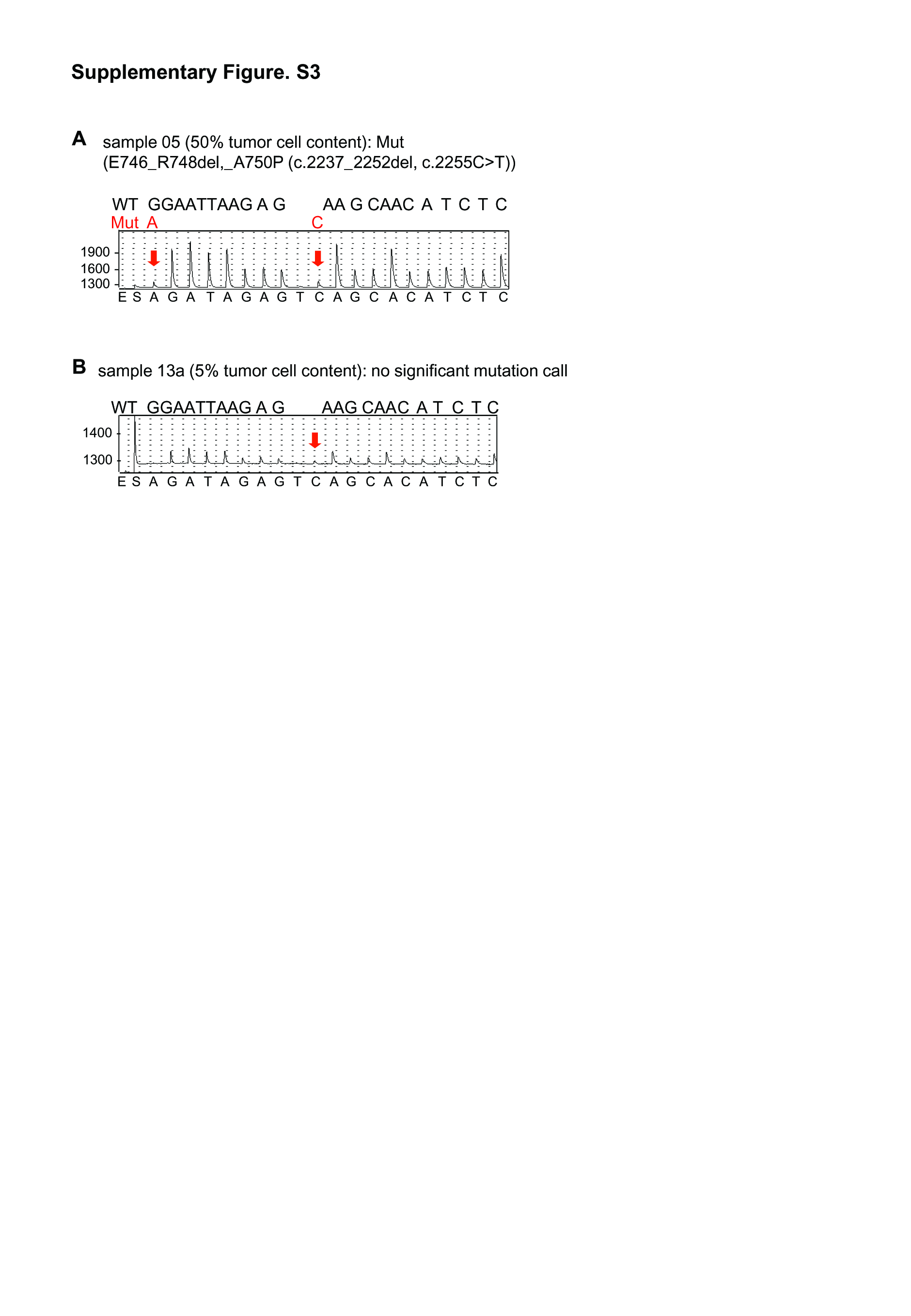

Supplement: Figure S3 — Pyrosequencing analysis of EGFR exon 19 in NSCLC tumor samples. (A) EGFR exon 19 deletion identified in sample with 50% tumor cell content that was previously not identified by dideoxy sequencing; (B) No significant mutation detection in sample 13a with a 5% tumor cell content. Expected position of mutation specific signals is marked by red arrow. del, deletion; Mut, mutation; WT, wild-type. (TIF) [file pone.0019601.s003.tif]

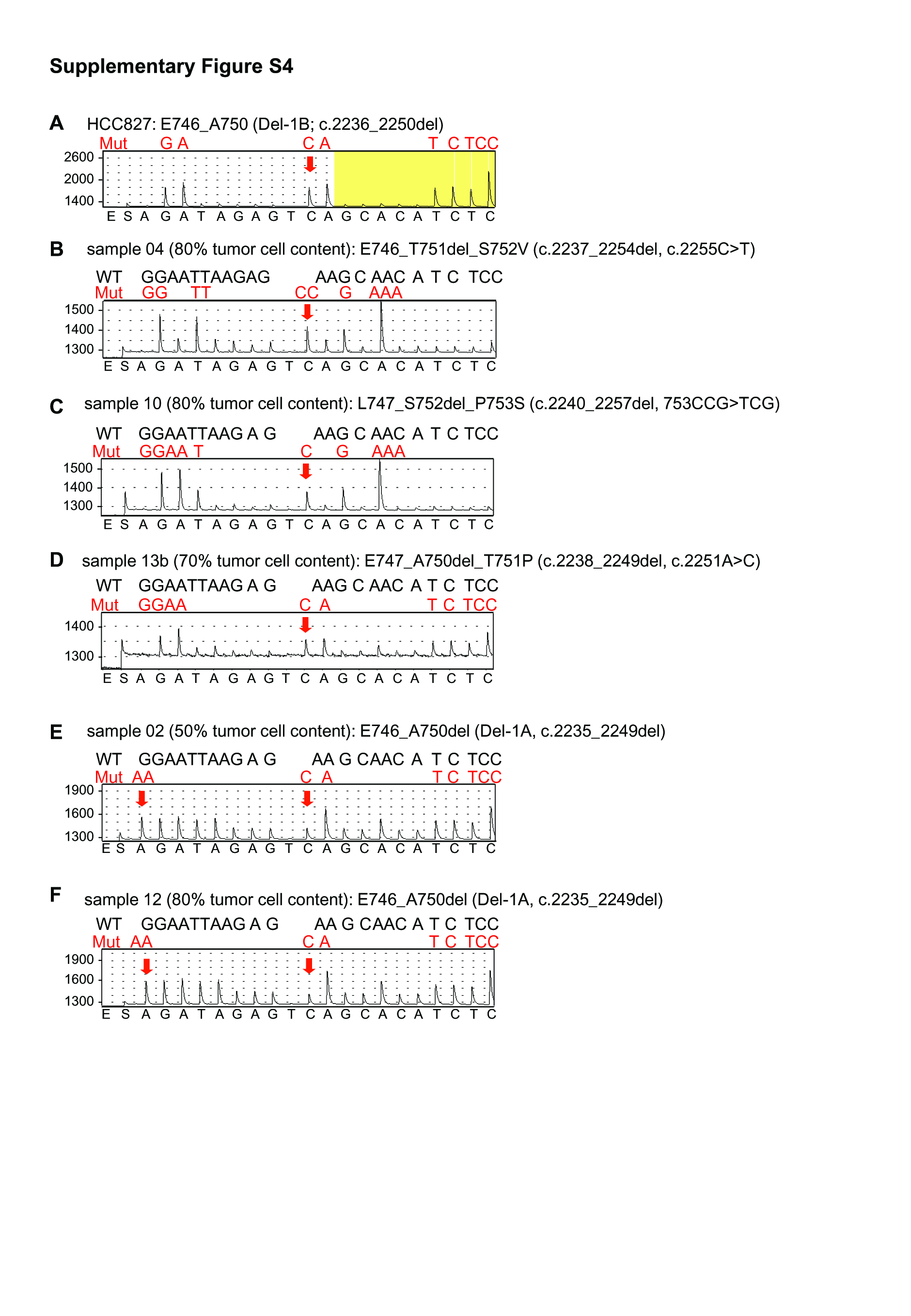

Supplement: Figure S4 — Pyrograms of EGFR exon 19 mutant NSCLC cell line and tumor samples. (A) Del-B mutation in cell line HCC827; (B–D) Tumor specimens with an high tumor cell content enabling precise characterisation of the individual mutation; (E, F) Tumor specimens with a moderate (50% and 80%) tumor cell content resulting in overlapping signals of wild-type and mutant alleles. Mutation specific signals are marked by red arrows. del, deletion; Mut, mutation; WT, wild-type. (TIF) [file pone.0019601.s004.tif]

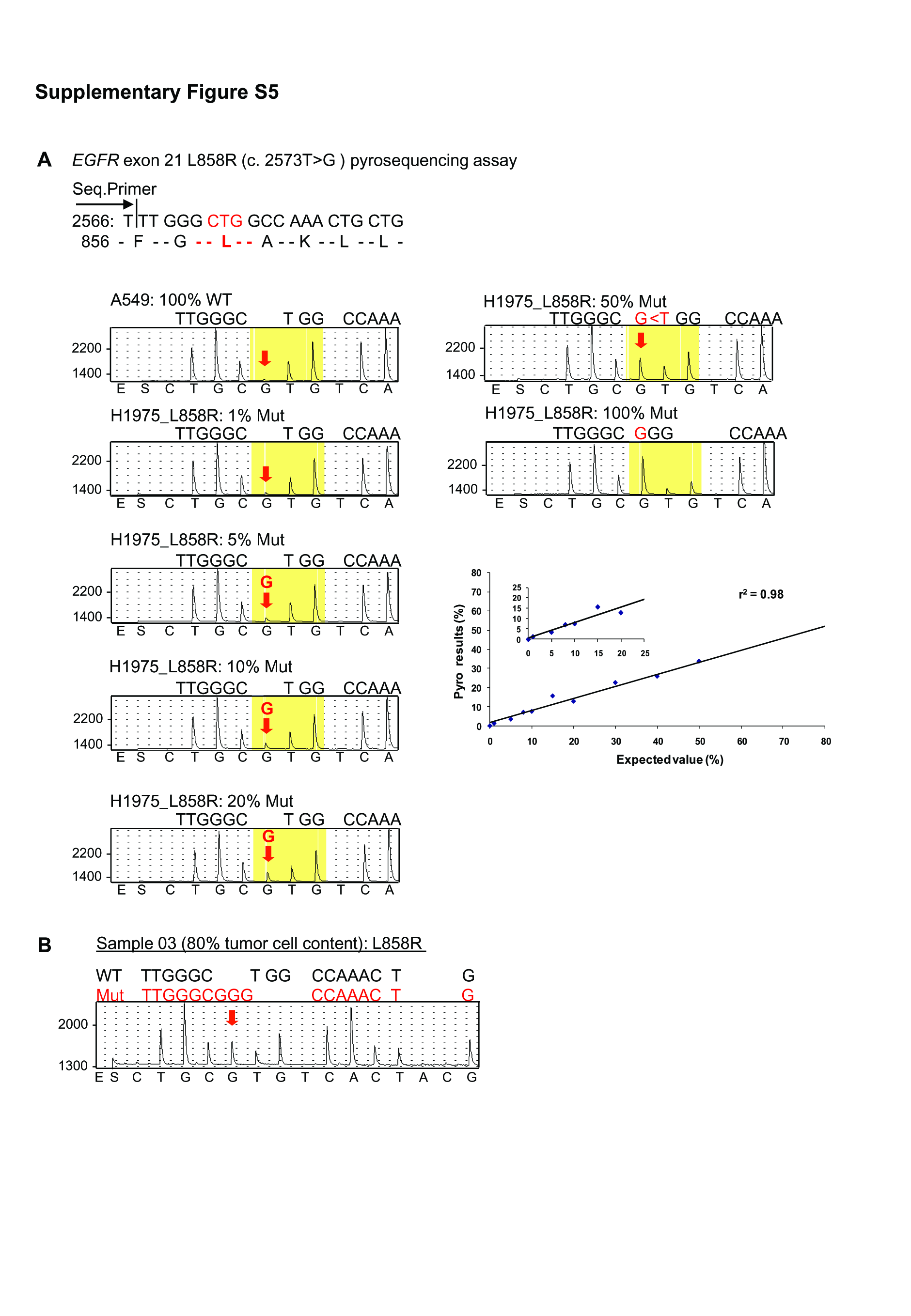

Supplement: Figure S5 — Sensitivity and linearity testing of the EGFR exon 21 (L858R) pyrosequencing assay. (A) Mixture study of PCR products from wild-type and L858R mutant NSCLC cell lines to determine the assay sensitivity limit of 5%–10%; (B) L858R point mutation identified in sample 03 (80% tumor cell content). Mutation specific signals are marked by red arrows. Mut, mutation; WT, wild-type. (TIF) [file pone.0019601.s005.tif]

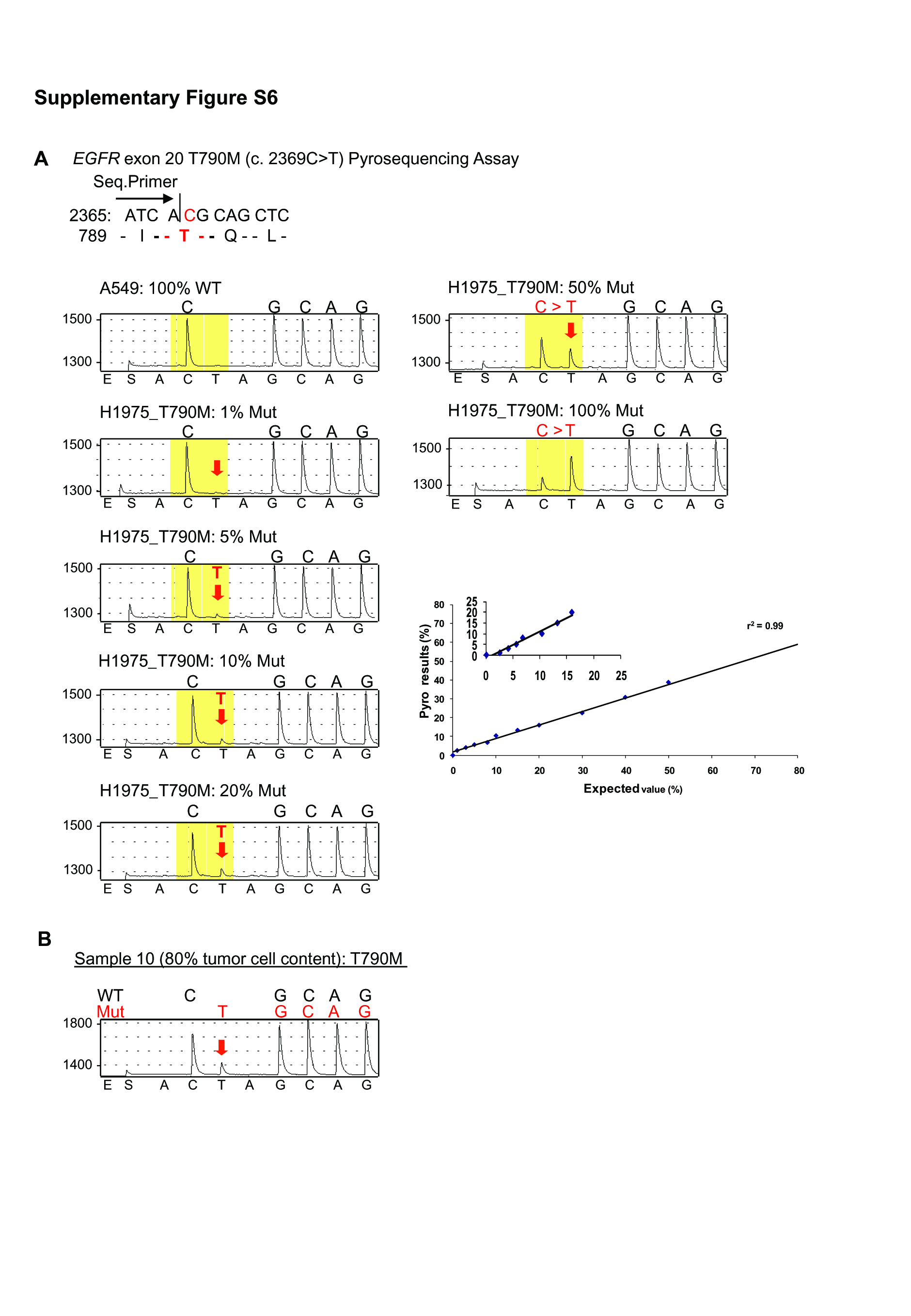

Supplement: Figure S6 — Sensitivity and linearity testing of the EGFR exon 20 (T790M) pyrosequencing assay. (A) Mixture study of PCR products from wild-type and T790M mutant NSCLC cell lines to determine the assay sensitivity limit of 5%–10%; (B) T790M point mutation detected in sample 10 (80% tumor cell content) that was previously not detected by conventional dideoxy sequencing. Mutation specific signals are marked by red arrows. Mut, mutation; WT, wild-type. (TIF) [file pone.0019601.s006.tif]

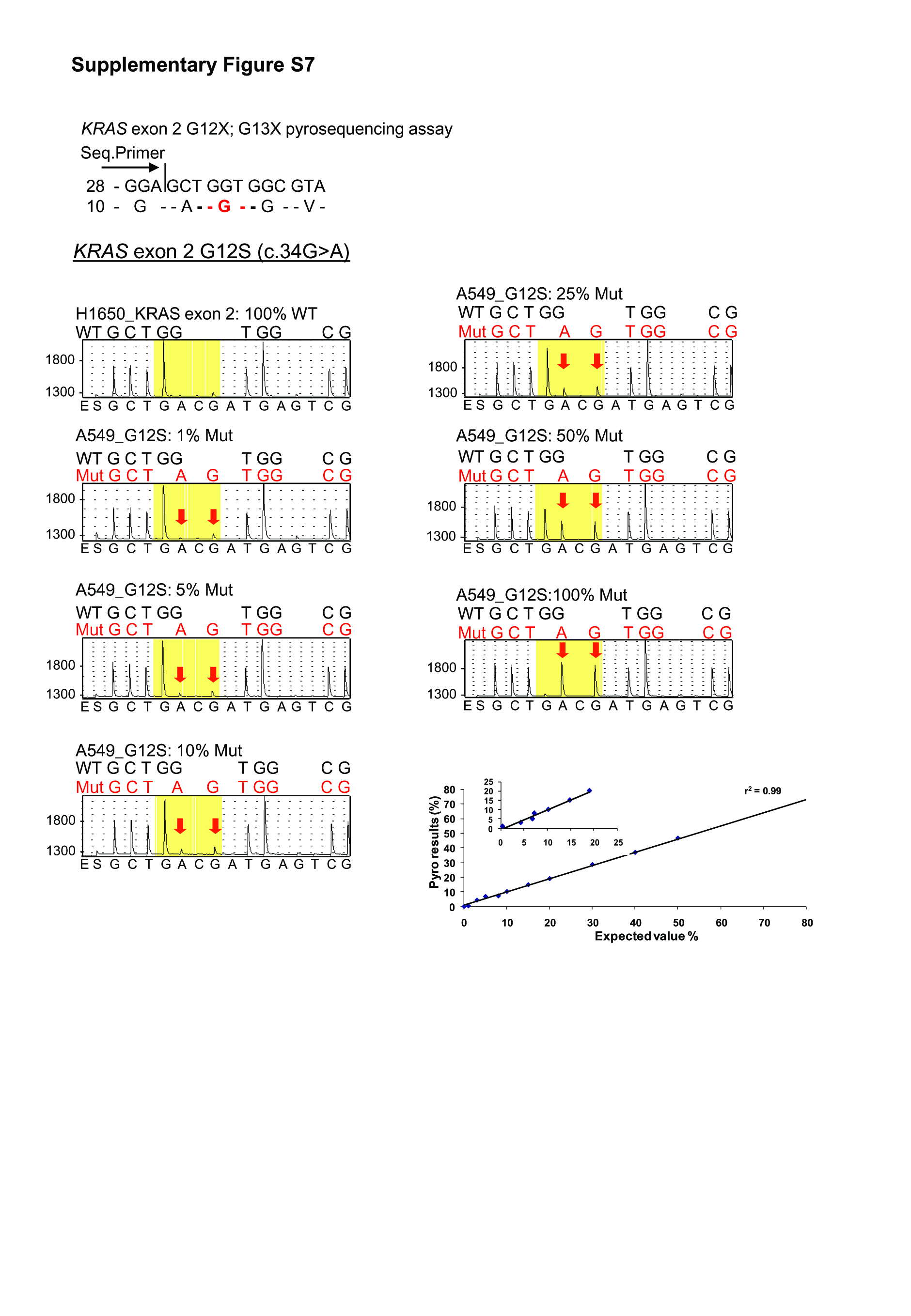

Supplement: Figure S7 — Sensitivity and linearity testing of the KRAS exon 2 (G12X;G13X) pyrosequencing assay. Mixture study of PCR products from wild-type and G12S KRAS exon 2 mutant NSLC cell lines to determine the assay sensitivity limit 0f 5%–10%. Mutation specific signals are marked by red arrows. Mut, mutation; WT, wild-type. (TIF) [file pone.0019601.s007.tif]

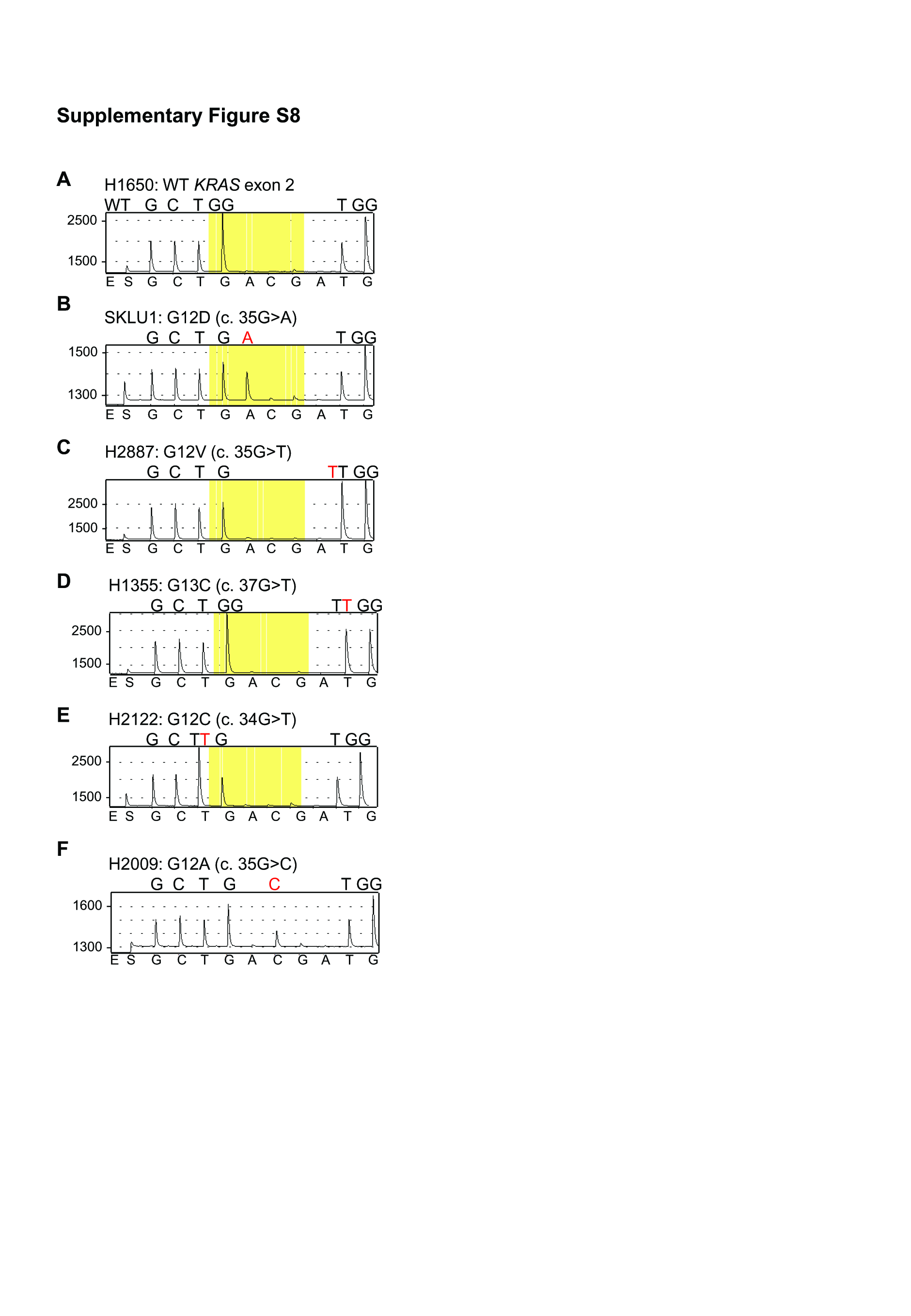

Supplement: Figure S8 — Pyrograms of KRAS exon 2 mutant NSCLC cell lines. (A) wild-type KRAS in H1650; (B) G12D in SKLU1; (C) G12V in H2887; (D) G13C in H1355; (E) G12C in H2122; (F) G12A in H2009. WT, wild-type. (TIF) [file pone.0019601.s008.tif]

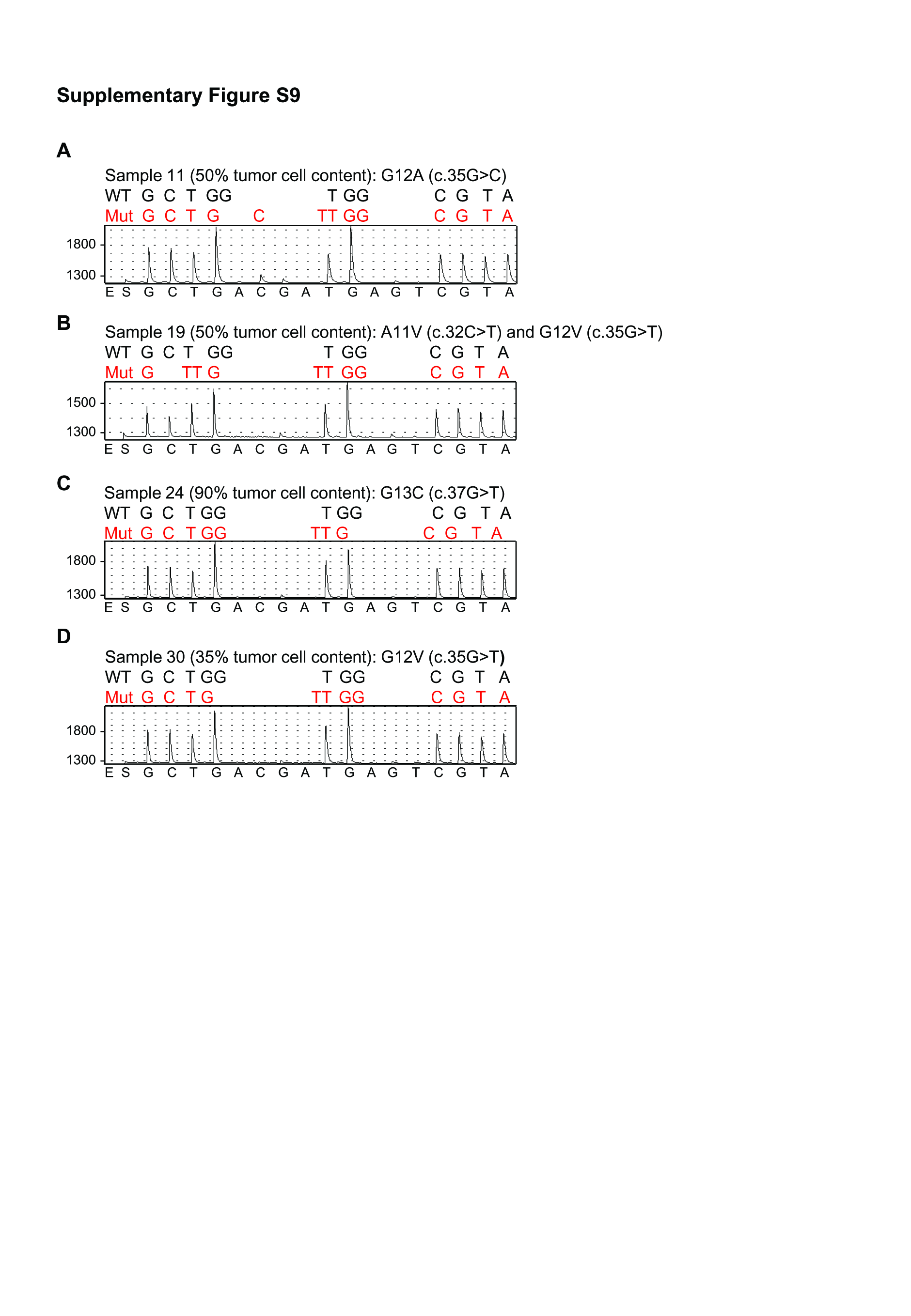

Supplement: Figure S9 — Pyrograms of NSCLC tumor samples harbouring different KRAS mutations. (A) G12A in sample 11; (B) A11V, G12V in sample 19; (C) G13C in sample 30 and (D) G12V in sample 30. Mut, mutation; WT, wild-type. (TIF) [file pone.0019601.s009.tif]

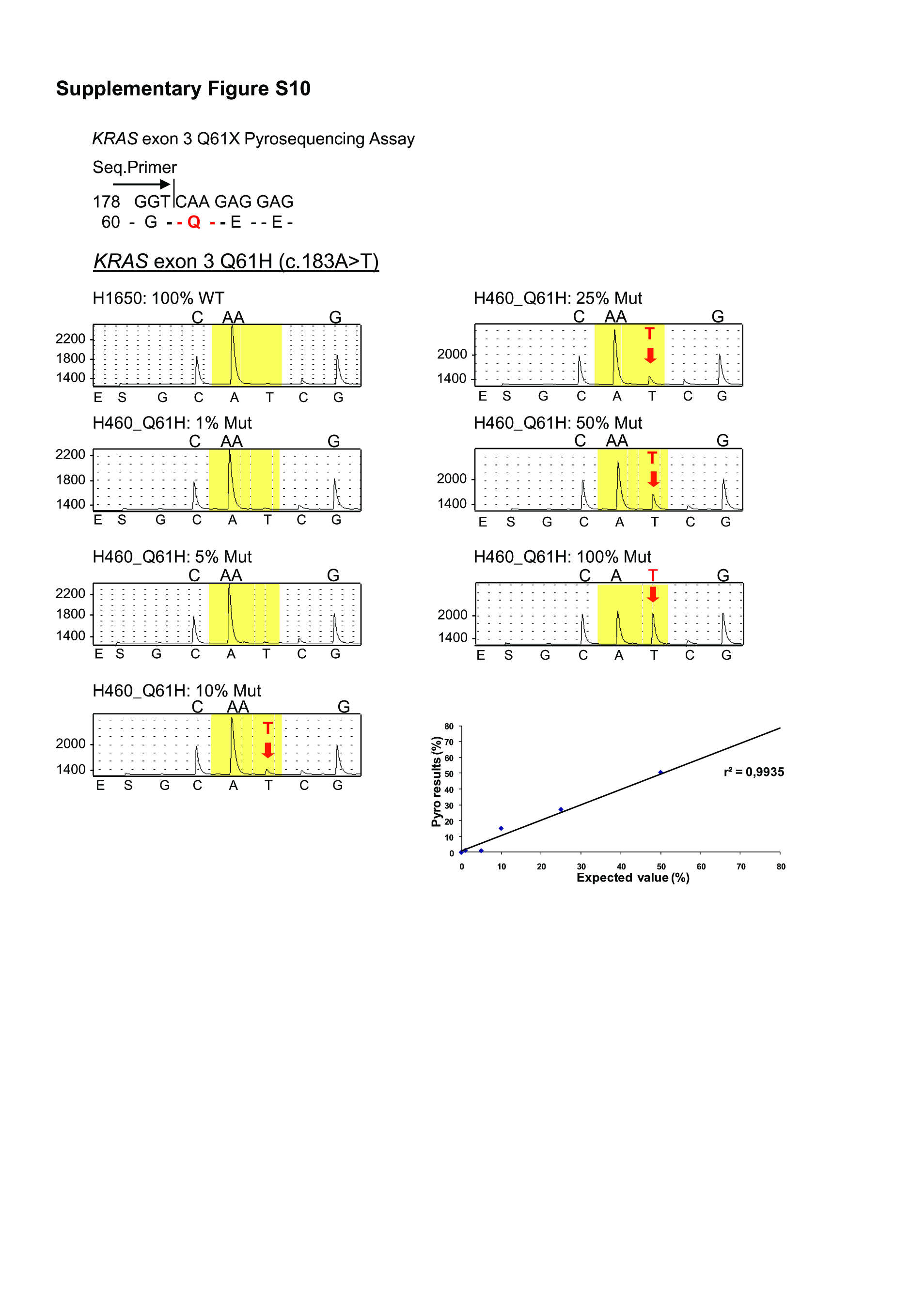

Supplement: Figure S10 — Sensitivity and linearity testing of the KRAS exon 3 (Q61X) pyrosequencing assay. Mixture study of PCR products from wild-type and Q61H KRAS exon 3 mutant NSCLC cell lines to determine the assay sensitivity limit of 10%. Mutation specific signals are marked by red arrows. Mut, mutation; WT, wild-type. (TIF) [file pone.0019601.s010.tif]

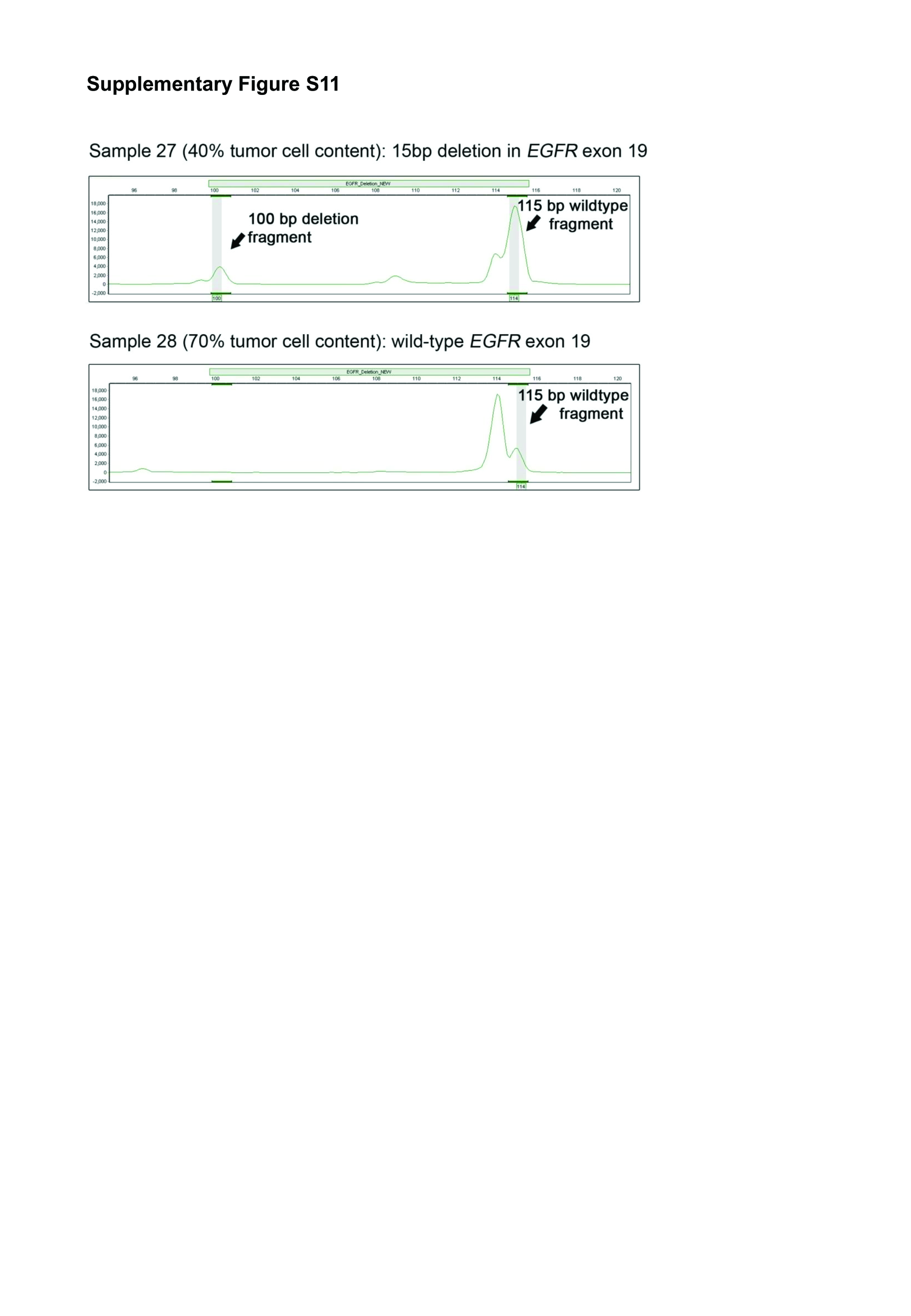

Supplement: Figure S11 — Fragment length analysis of EGFR exon 19 PCR products. (A) sample 27 harbouring a 15 bp deletion (100 bp fragment); (B) wild-type EGFR exon 19 (115 bp fragment) of sample 28. (TIF) [file pone.0019601.s011.tif]
