# Supplementary material for: Benchmarking of Mutation Diagnostics in Clinical Lung Cancer Specimens
Source: PLoS One. 2011 May 5;6(5):e19601. doi: 10.1371/journal.pone.0019601 (PMC3088700; doi:10.1371/journal.pone.0019601)
Supplement: Figure S12 — Read frequency of T790M mutation in pre-treatment tumor specimens. Depicted is the average read frequency of T790M separately for EGFR wildtype and EGFR mutated tumor specimens. Tumor specimen 10b with a high allele frequency of T790M and clinical resistance to erlotinib treatment is not shown in this diagram. (PPT) [file pone.0019601.s012.ppt]

## Slide 1
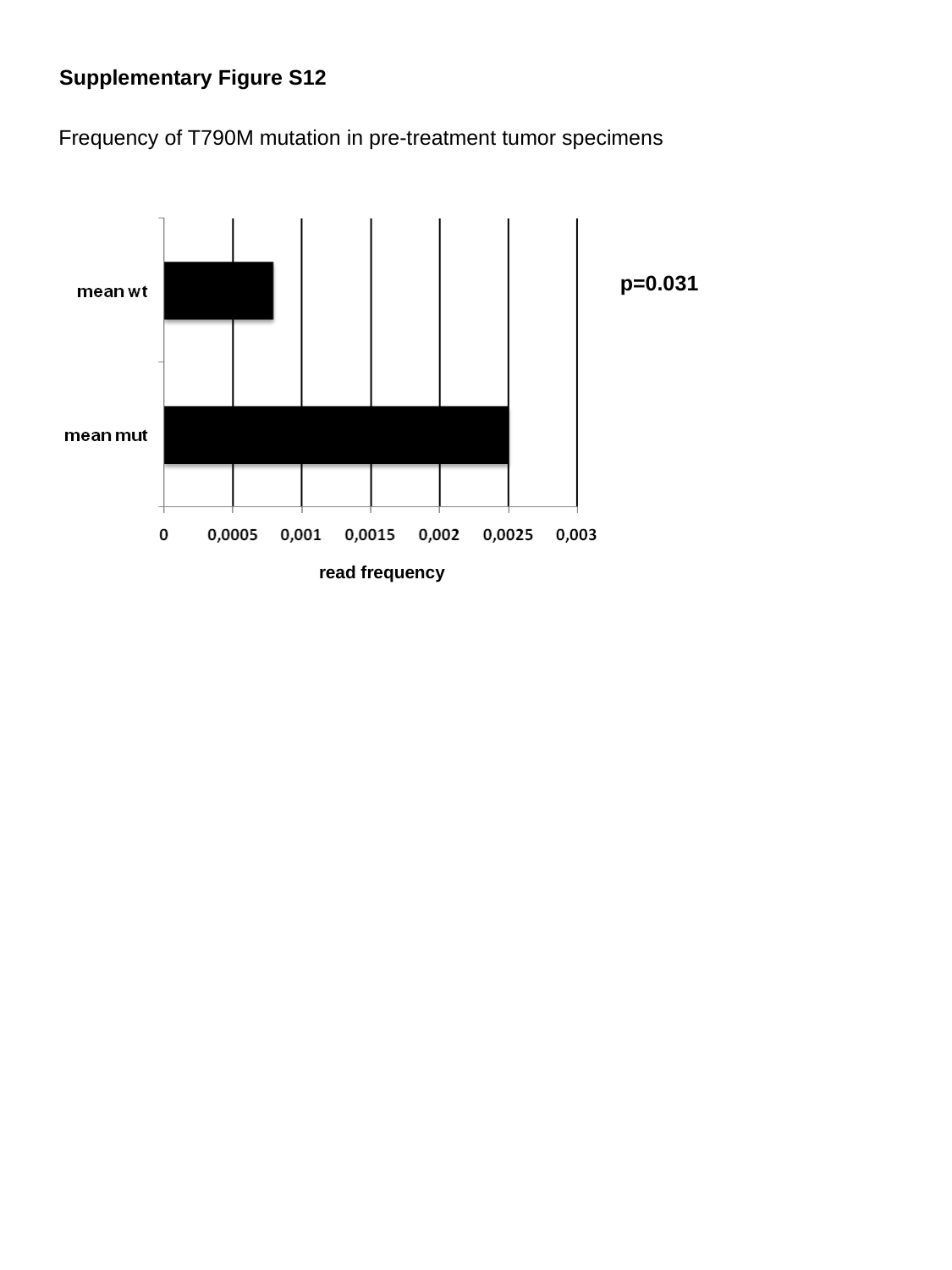

Supplementary Figure S12
Frequency of T790M mutation in pre-treatment tumor specimens
p=0.031
read frequency
