# Supplementary material for: Benchmarking of Mutation Diagnostics in Clinical Lung Cancer Specimens
Source: PLoS One. 2011 May 5;6(5):e19601. doi: 10.1371/journal.pone.0019601 (PMC3088700; doi:10.1371/journal.pone.0019601)
Supplement: Table S1 — EGFR and KRAS mutant NSCLC cell lines. NSCLC cell lines harbouring different EGFR and KRAS mutations with respective amino acid changes are shown. (DOC) [file pone.0019601.s013.doc]

**Supplementary Table S1.** *EGFR* and *KRAS* mutant NSCLC cell lines

| Cell line | Gene locus | Nucleotide | Aminoacid |
| --- | --- | --- | --- |
| H1650 | EGFR exon 19 | 2235_2249del | E746_A750 (Del-1a) |
| HCC827 | EGFR exon 19 | 2236_2250del | E746_A750 (Del-1b) |
| H1975 | EGFR exon 20 and 21 | c.2369C>T; c.2573T>G | T790M; L858R |
| H2009 | KRAS exon 2 | c.35G>C | G12A |
| H2122 | KRAS exon 2 | c.34_36GGT>TGC | G12C |
| SKLU1 | KRAS exon 2 | c.35G>A | G12D |
| A549 | KRAS exon 2 | c.34>A | G12S |
| H2887 | KRAS exon 2 | c.35_36GT>TC | G12V |
| H1355 | KRAS exon 2 | c.37G>T | G13C |
| H460 | KRAS exon 3 | 183A>T | Q61H |
