# Supplementary material for: Benchmarking of Mutation Diagnostics in Clinical Lung Cancer Specimens
Source: PLoS One. 2011 May 5;6(5):e19601. doi: 10.1371/journal.pone.0019601 (PMC3088700; doi:10.1371/journal.pone.0019601)
Supplement: Table S2 — Primer sequences for EGFR and KRAS nested PCR. External and internal primer sequences used for EGFR exon18–21 and KRAS exon 2 and 3 PCR are shown. (DOC) [file pone.0019601.s014.doc]

**Supplementary Table S2.** Primer sequences for *EGFR* and *KRAS* nested PCR

| **Primer Name** | **Forward Sequence** | **Reverse Sequence** |
| --- | --- | --- |
| EGFR exon18 external | ATGCCGTGGCTGCTGGTC | CCCACCAGACCATGAGAGG |
| EGFR exon 18 internal | CCATGTCTGGCACTGCTTTC | CCTGTGCCGGGACCTTAC |
| EGFR exon 19 external | GCTGGTAACATCCACCCAGAT | GCAGGGTCTAGAGCAGAGCAG |
| EGFR exon 19 internal | TGCCAGTTAACGTCTTCCTTCTC | AAAGGTGGGCCTGAGGTT |
| EGFR exon 20 external | CCTCCTTCTGGCCACCAT | GCAGACCGCATGTGAGGAT |
| EGFR exon 20 internal | AGCCACACTGACGTGCCTCT | CCGTATCTCCCTTCCCTGATTA |
| EGFR exon 21 external | GGCATGAACATGACCCTGAAT | CAGCCTGGTCCCTGGTGT |
| EGFR exon 21 internal | ATGCAGAGCTTCTTCCCATGA | GCTGACCTAAAGCCACCTCCT |
| KRAS exon 2 external | CGTCTGCAGTCAACTGGAATTT | AAAGAATGGTCCTGCACCAGTAA |
| KRAS exon 2 internal | TTAACCTTATGTGTGACATGTTCTAA | TGGTCCTGCACCAGTAATATGC |
| KRAS exon 3 external | TGCACTGTAATAATCCAGACTGTG | GCATGGCATTAGCAAAGACTCA |
| KRAS exon 3 internal | CTGTAATAATCCAGACTGTGTTTCTC | GCATTAGCAAAGACTCAAAAA |
| M13 | GTAAAACGACGGCCAGT | CAGGAAACAGCTATGACC |
