# Supplementary material for: Benchmarking of Mutation Diagnostics in Clinical Lung Cancer Specimens
Source: PLoS One. 2011 May 5;6(5):e19601. doi: 10.1371/journal.pone.0019601 (PMC3088700; doi:10.1371/journal.pone.0019601)
Supplement: Table S3 — Primer sequences for EGFR and KRAS pyrosequencing. Shown are primer sequences for five different pyrosequencing assays for sequencing analysis of EGFR exon 19–21 and KRAS exon 2 and 3. (DOC) [file pone.0019601.s015.doc]

**Supplementary Table S3.** Primer sequences for EGFR and KRAS pyrosequencing

| **Assay** | **Name** | **Sequence** |
| --- | --- | --- |
| **EGFR exon 19** | Forward PCR primer | TAAAATTCCCGTCGCTATCAA |
|  | Reverse PCR primer | AGCTGCCAGACATGAGAAAAG |
|  | Sequencing primer | TTCCCGTCGCTATCAA |
|  | Dispensation order | AGATAGAGTCAGCACATCTCGA |
| **EGFR exon 20** | Forward PCR primer | CTGGGCATCTGCCTCACCT |
|  | Reverse PCR primer | TGTGTTCCCGGACATAGTCCA |
|  | Sequencing Primer | ACCGTGCAGCTCATCA |
|  | Dispensation order | ACTAGCAGC |
| **EGFR exon 21** | Forward PCR primer | AAACACCGCAGCATGTCAA |
|  | Reverse PCR primer | TCTTTCTCTTCCGCACCCA |
|  | Sequencing Primer | CATGTCAAGATCACAGATT |
|  | Dispensation order | CTGCGTGTCACTACG |
| **KRAS exon 2** | Forward PCR primer | CTGAATATAAACTTGTGGTAGTTG |
|  | Reverse PCR primer | TGTATCGTCAAGGCACTCT |
|  | Sequencing Primer | AAACTTGTGGTAGTTGGA |
|  | Dispensation order | GCTGACGATGAGTCGTA |
| **KRAS exon 3** | Forward PCR primer | GATGGAGAAACCTGTCTCTTGG |
|  | Reverse PCR primer | CCCTCCCCAGTCCTCATGTA |
|  | Sequencing Primer | TCTCGACACAGCAGGT |
|  | Dispensation Order | CGATGCATGAG |
