# Supplementary material for: Benchmarking of Mutation Diagnostics in Clinical Lung Cancer Specimens
Source: PLoS One. 2011 May 5;6(5):e19601. doi: 10.1371/journal.pone.0019601 (PMC3088700; doi:10.1371/journal.pone.0019601)
Supplement: Table S4 — EGFR and KRAS gene specific primers for massively parallel sequencing. Primer pairs for massively parallel sequencing of EGFR exon 18–21 and KRAS exon 2 and 3 are depicted. (DOC) [file pone.0019601.s016.doc]

**Supplementary Table S4.** *EGFR* and *KRAS* gene specific primers for massively parallel sequencing

| **Primer name** | **Forward Sequence** | **Reverse Sequence** |
| --- | --- | --- |
| EGFR exon 18 | GGCACTGCTTTCCAGCATGGT | CCTGTGCCGGGACCTTAC |
| EGFR exon 19 | TGCCAGTTAACGTCTTCCTTC | AAAGGTGGGCCTGAGGTT |
| EGFR exon 20 | CGAAGCCACACTGACGTGCCT | CCGTATCTCCCTTCCCTGAT |
| EGFR exon 21 | TCCCTCACAGCAGGGTCTTCT | GGCTGACCTAAAGCCACCT |
| KRAS exon 2 | CATTATTTTTATTATAAGGCCT | AGAATGGTCCTGCACCAGTAA |
| KRAS exon 3 | TGTTTCTCCCTTCTCAGGATT | TTCAATTTAAACCCACCTATAATG |
