# Supplementary material for: Benchmarking of Mutation Diagnostics in Clinical Lung Cancer Specimens
Source: PLoS One. 2011 May 5;6(5):e19601. doi: 10.1371/journal.pone.0019601 (PMC3088700; doi:10.1371/journal.pone.0019601)
Supplement: Table S5 — Comparison of mutation detection performance between sequencing methods. Sensitivity of EGFR and KRAS mutation detection by three different sequencing technologies (dideoxy sequencing, pyrosequencing, parallel sequencing) is shown. (DOC) [file pone.0019601.s017.doc]

**Supplementary Table S5.** Comparison of mutation detection performance between sequencing methods

| **N = 24 samples** | **Dideoxy Sequencing** | **Pyrosequencing** | **Parallel Sequencing** |
| --- | --- | --- | --- |
| *EGFR* mutations | 9 | 12 | 14 |
| *KRAS* mutations | 3 | 4 | 4 |
| Total mutations | 12 | 16 | 18 |
| False negative | 6 (33 %) | 2 (11 %) | 0 |
| Sensitivity rate | 67 % (12/18 mutations) | 89 % (16/18 mutations) | 100 % (18/18 mutations) |
